# ^18^F-Fluorodeoxyglucose Positron Emission Tomography/Computed Tomography Accuracy in the Staging of Non-Small Cell Lung Cancer: Review and Cost-Effectiveness

**DOI:** 10.1155/2014/135934

**Published:** 2014-11-05

**Authors:** Nieves Gómez León, Sofía Escalona, Beatriz Bandrés, Cristobal Belda, Daniel Callejo, Juan Antonio Blasco

**Affiliations:** ^1^Department of Radiology, Research Institute La Princesa, La Princesa University Hospital, C/Diego de León 62, 28006 Madrid, Spain; ^2^Health Technology Assessment Unit, Lain Entralgo Agency, C/Gran Vía 27, 28013 Madrid, Spain; ^3^National School of Health, Sinesio Delgado 4, 28029 Madrid, Spain; ^4^Institute for Health and Consumer Protection, Joint Research Centre, European Commission, Via E. Fermi 2749, 21027 Ispra, Italy

## Abstract

Aim of the performed clinical study was to compare the accuracy and cost-effectiveness of PET/CT in the staging of non-small cell lung cancer (NSCLC). *Material and Methods*. Cross-sectional and prospective study including 103 patients with histologically confirmed NSCLC. All patients were examined using PET/CT with intravenous contrast medium. Those with disease stage ≤IIB underwent surgery (*n* = 40). Disease stage was confirmed based on histology results, which were compared with those of PET/CT and positron emission tomography (PET) and computed tomography (CT) separately. 63 patients classified with ≥IIIA disease stage by PET/CT did not undergo surgery. The cost-effectiveness of PET/CT for disease classification was examined using a decision tree analysis. *Results*. Compared with histology, the accuracy of PET/CT for disease staging has a positive predictive value of 80%, a negative predictive value of 95%, a sensitivity of 94%, and a specificity of 82%. For PET alone, these values are 53%, 66%, 60%, and 50%, whereas for CT alone they are 68%, 86%, 76%, and 72%, respectively. Incremental cost-effectiveness of PET/CT over CT alone was €17,412 quality-adjusted life-year (QALY). *Conclusion*. In our clinical study, PET/CT using intravenous contrast medium was an accurate and cost-effective method for staging of patients with NSCLC.

## 1. Introduction

Lung cancer is the most common fatal neoplasm in developed countries. Non-small cell lung cancer (NSCLC) is responsible for 80% of all deaths from this neoplasm [1]. Despite all efforts to improve early diagnosis, survival rates remain very low (about 18% at 5 years) [2]. The stage at which NSCLC is detected is the most important of all prognostic factors and the only one that determined treatment options in our study before targeted therapy was administered. The TNM classification of malignant tumors [3] for staging NSCLC is internationally accepted and validated.

CT is currently the most commonly used technique in tumor staging. However, results, which are based on normal lymph node size, have limited value. In contrast, PET provides valuable functional information because it can detect metabolically active tumor cells.

Over the last 10 years, PET has become an important tool for staging lung cancer, given its high sensitivity in the detection of lymph node involvement and distant metastasis [4] and its current use in presurgical stratification of NSCLC [5]. The combination of CT and PET makes it possible to visualize anatomical and metabolic images together, thus minimising the limitations and maximising the benefits of each technique individually.

The aims of the present study were as follows: (1) to compare the accuracy of PET/CT and of PET and CT alone for staging NSCLC, with histological examination as the gold standard; and (2) to determine the cost-effectiveness of PET/CT in staging of NSCLC.

## 2. Material and Methods

### 2.1. Patient Sample

This prospective study involved 103 patients with a clinical and histological diagnosis of NSCLC (confirmed by fine-needle aspiration and biopsy of the tumor) whose disease stage was not known and who had not yet received treatment.

All patients gave their written informed consent to participate in the study in accordance with the regulations of the institutional review board.

The patients' medical histories were examined before decisions on treatment were taken. Demographic data, histological diagnosis, and clinical status were recorded, and all patients underwent PET/CT.

### 2.2. PET/CT Protocol

All images were acquired with a combined in-line PET/CT system (Discovery LS; GE Healthcare, Milwaukee, Wisconsin, USA) that integrates a 4-detector row spiral CT (LightSpeed Plus; GE Healthcare) with a PET scanner (Advance NXi; GE Healthcare).

A standard dose of 370 MBq of ^18^F-FDG was intravenously injected 45–60 minutes before imaging. Scanning was performed from the base of the skull to the midthigh while patients were in the supine position. To obtain a precise anatomic correlation between PET and CT images, whole-body scanning was performed with the arms in the upright position for both PET and CT.

Diagnostic contrast-enhanced CT was initially performed at 140 KV and with automatic current adjustment (maximum, 300 mA) according to the patient's weight.

A volume of 140 mL of iodinated contrast agent (iobitridol[Xenetix 300], 300 mg of iodine per milliliter; Guerbet) was first administered intravenously at 3 mL/s using an automated injector (model XD 5500; Ulrich Medical Systems). PET emission scanning was performed immediately after CT, with an identical transverse field of view and in the caudocranial direction.

Coregistered scans were displayed using Entegra or Xeleris software (GE Healthcare).

### 2.3. Interpretation of Isolated PET Images

A specialist in nuclear medicine interpreted PET images. Abnormal ^18^F-FDG uptake was defined as accumulation outside the normal anatomic structures and of greater intensity than background activity inside the normal structures. Any visual focus of  ^18^F-FDG uptake over that of the background was deemed to represent tumor tissue.

The uptake of the radiotracer was also assessed semiquantitatively using the standardized uptake value (SUV) method.

However, the SUV is also limited in that it is affected by many factors, including blood sugar level, body weight, time elapsed since administration of the radiotracer, and the size and heterogeneity of the area of interest. Most authors, therefore, agree with visual interpretation of the results, although an SUV higher than 2.5/3 is considered pathological [14]. Currently, we apply the recommendations described by the Dutch F18-FDG-PET standard (NEDPAS). A conclusion was recorded for each patient in agreement with the TNM classification system.

### 2.4. Interpretation of Isolated CT Images

CT images were interpreted by a radiologist. Lymph nodes with a shorter axis >10 mm were deemed positive. Chest lymph nodes were located according to the criteria of the American Thoracic Society [15].

### 2.5. Interpretation of Combined PET and CT Images

PET/CT images were assessed by the nuclear medicine specialist and radiologist working as a team. Lymph nodes were considered diseased when they showed pathological ^18^F-FDG activity, irrespective of their size. Those showing no such activity were considered disease-free. The patient was considered to have extranodal disease when ^18^F-FDG activity in the tumor was greater than that of the background organ and the SUV was higher than 2.5/3.

### 2.6. Classification of Disease by PET/CT, CT, and PET

Disease stage was assigned according to the PET/CT results and to the PET and CT results taken separately following the criteria of the 7th edition of the TNM system [3].

Patients were then classified as candidates for surgery (stage ≤IIB) or for other treatments (≥IIIA).

### 2.7. Classification of Disease by Histological Examination

All primary lung tumors of the 103 patients were studied histologically, either by bronchoscopy or CT-guided fine needle aspiration.

A histological evaluation was performed for the tumor samples of all 103 patients (43 patients with adenocarcinoma, 29 with large-cell carcinoma, and 31 with epidermoid carcinoma). Of these patients, only 40 were candidates for surgery.

### 2.8. Gold Standard Reference

Mediastinoscopy was carried out during surgery, and each node or lesion was histologically confirmed. If the stage was IIIB and/or IV, the gold standard was also the histological confirmation in samples taken using biopsy, CT-guided PAAF, and/or fibrobronchoscopy, as in the case of surgical patients. Mediastinal nodes were not studied when the result had no impact on treatment decisions. Endoscopic ultrasound (EUS) and endobronchial ultrasound (EBUS) are used to reach mediastinal nodes. EUS involves upper gastrointestinal endoscopy and enables visualization and sampling of the posterior mediastinal nodes. A similar ultrasound system is used in bronchoscopy, with which most of the mediastinal nodes are accessible. However, at the time our patients were recruited these techniques were not available at our hospital. A 5-year clinical follow-up revealed that no stage IIIB and IV patients survived.

In cases with suspicion of adrenal metastasis, other tests (e.g., magnetic resonance imaging) were performed and biopsy specimens were taken. All suspicious lesions in the liver or bone were biopsied. Patients were treated with chemotherapy and/or radiotherapy.

### 2.9. Interpretation of Results: Statistical Analysis

All calculations were performed using IBM SPSS v.19.0 software. Significance was set at *P* < 0.05.

Kappa indices were determined to estimate the degree of agreement between the staging results of the histological examination and those of PET/CT, CT alone, and PET alone for the different subgroups of patients and tumors.

### 2.10. Cost-Effectiveness

A cost-effectiveness analysis was performed to determine which method (PET/CT, PET, or CT) should be the approach of choice [16] for staging of NSCLC and to select the treatment strategy [17].

**Figure 1:**
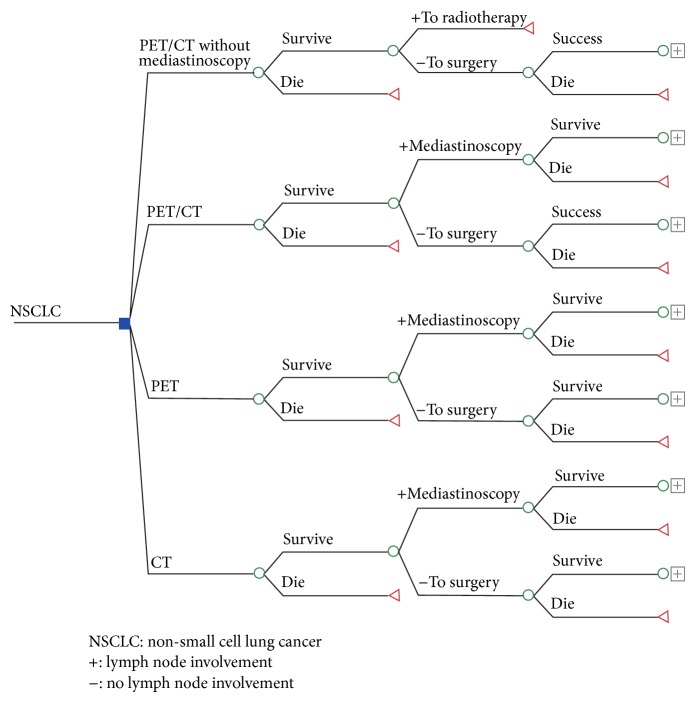
Structure of the decision tree for assessing the cost-effectiveness of the different staging alternatives examined.

A decision tree model (Figure 1) was constructed for determination of disease stage in patients with NSCLC using each of the three techniques [18].

Health benefits for patients were summarized as quality-adjusted life-years (QALYs). Patient survival was predicted according to disease stage and treatment provided based on the criteria of the SEER Cancer Statistics Review [10].

The DEALE model [19] was used to calculate life expectancy from the 5-year survival value. Utilities used to estimate QALYs were retrieved from the literature [6]. The unit costs included in the model were taken from the official Spanish National Health System data for 2010 [8, 9] and are expressed in euros.

The variables used in the model are summarized in Table 1 [6–13].

### 2.11. Economic Sensitivity Analysis

The sensitivity analysis also included a branch in the decision tree that considered those treatment decisions based exclusively on PET/CT data (i.e., without histological confirmation).

Once the value for the base case (QALYs) was calculated, univariate sensitivity analysis was performed [20] to identify the effect of uncertainty on the values of the different variables in the decision tree. The upper and lower limits of the 95% confidence intervals for these values were calculated. Costs were included at ±20% of their value (Table 1).

A probabilistic sensitivity analysis based on 10,000 Monte Carlo simulations was performed to analyze the joint effect of uncertainty on the values of the variables included in the decision tree. The most commonly reported distributions of the values were used in all calculations [21].

## 3. Results

The study included 103 patients (90 men [87.4%] and 13 women [12.6%]) with a mean age of 68 years (SD 10, range 46 to 83). The initial histological analysis revealed that 41 patients had adenocarcinoma, two had bronchoalveolar carcinoma, 31 had epidermoid carcinoma, and 29 had large-cell carcinoma.

**Figure 2:**
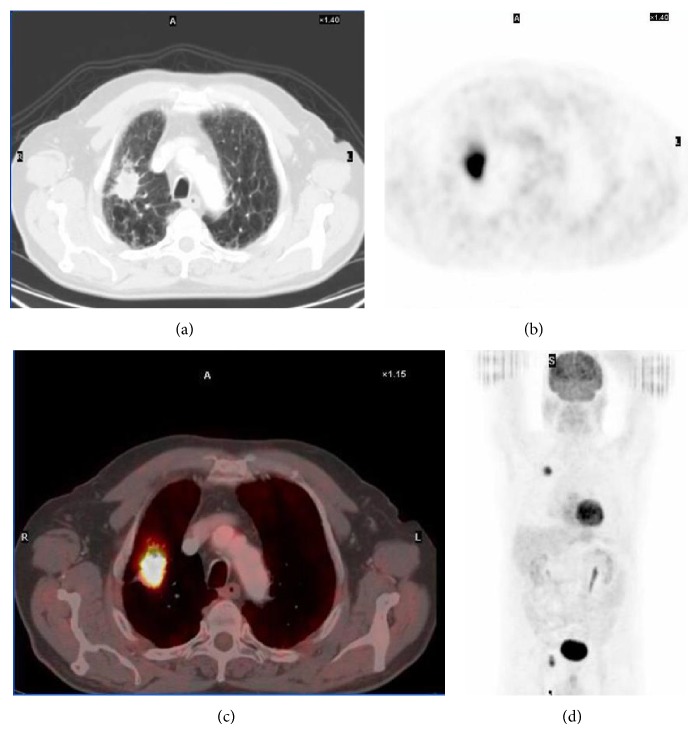
Images for a 60-year-old man. (a) CT image of an NSCLC primary tumor (epidermoid carcinoma) in the right upper lobe. (b) PET image showing intense ^18^F-FDG uptake. (c) PET/CT image showing tumor localisation and radiotracer uptake. (d) Coronal whole-body PET image.

Forty of the 103 patients (38.8%, 36 men and four women) were classified by PET/CT and histology as candidates for surgery; nine of these patients had stage IA disease, 11 had stage IB disease, six had stage IIB disease,12 had stage IIIA disease, and two had stage IIIB disease (Figure 2).

**Figure 3:**
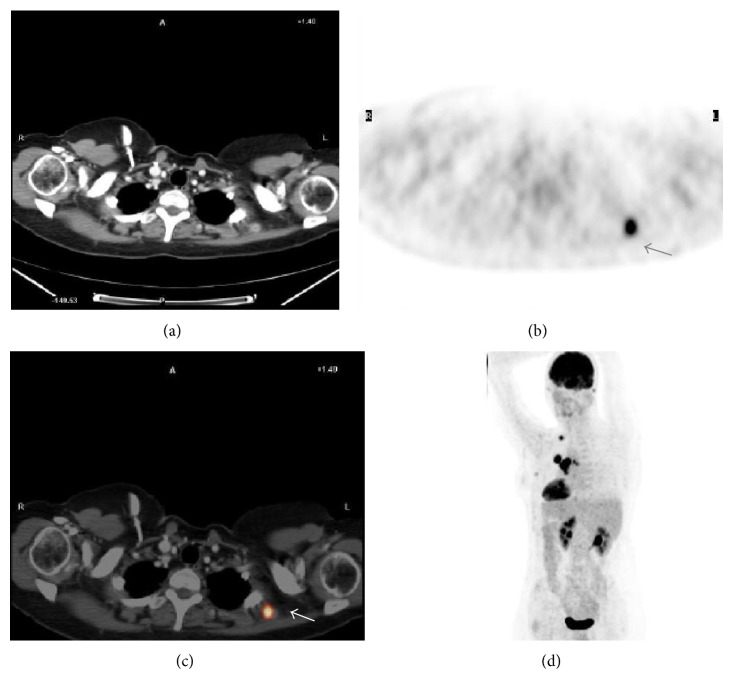
Images for a 55-year-old woman with stage IIIB NSCLC (adenocarcinoma). (a) CT imaging failed to detect any metastatic tumor. (b) PET image showing intense, nonlocalised uptake of ^18^F-FDG. (c) PET/CT image showing ^18^F-FDG uptake in a metastatic tumor in the trapezius muscle. Histological confirmation of the metastatic nature of the lesion. (d) Coronal whole-body PET image showing primary and metastatic lesions (arrowheads). PET = positron emission tomography; CT = computed tomography.

Twenty-three nonsurgical patients had stage IV (metastatic) disease (10 with adrenal gland involvement, one with trapezius muscle involvement (Figure 3), one with bone involvement, and 11 with multiple metastases, including liver involvement). An additional two suspicious bone biopsies revealed false-positive results; one was a posttraumatic injury, and the other was compatible with fibrous dysplasia.

### 3.1. TNM Staging Performance

The concordance of each diagnostic technique (CT, PET, and PET/CT) in the TNM staging of patients with a histologically proven stage is shown in Table 2. The concordance of each diagnostic technique with the final histology results for tumor size, nodes, and metastasis is shown in Table 3.

Compared to the histology results, PET/CT more accurately staged disease in all 103 patients (kappa = 0.83) than CT (kappa = 0.694) and PET (kappa = 0.614).

### 3.2. T Staging

Primary tumor (T) staging based on PET/CT data sets was more accurate than staging based on individual CT or PET (Table 4).

### 3.3. N Staging

Concordance for staging the lymph node involvement (N) between PET/CT and histological examination was good (kappa = 0.75, *P* < 0.001). Five patients were incorrectly classified (false positives) by PET/CT as having grade N1 (*n* = 4) or N2 (*n* = 1) lymph node involvement; histological examination yielded a classification of grade N0 involvement (these lymph nodes only showed anthracosis and/or lymphoid hyperplasia). Agreement between PET and histological examination was moderate (kappa = 0.57, *P* < 0.001). CT alone classified 18 patients incorrectly, thus showing very poor agreement with the histological classification.

Good concordance was observed between PET/CT and the final node assessment (kappa = 0.75, *P* < 0.001), thus indicating that PET/CT is the best of the three diagnostic techniques.

### 3.4. M Staging

Concordance for metastatic disease (M) classification observed between PET/CT and histological examination was good (kappa = 0.90, *P* < 0.001) when the latter was deemed medically necessary. Agreement between PET and histological examination was also good (kappa = 0.78, *P* < 0.001), as it was for CT alone (kappa = 0.81, *P* < 0.001). Compared with histological examination, PET/CT accurately staged disease in the 103 patients (kappa = 0.83); compared with CT (kappa = 0.694) and PET (kappa = 0.614). The two false-positive results recorded in two patients with bone involvement (see above) due to traumatic injury and fibrous dysplasia.

### 3.5. Overall Disease Staging Accuracy

Staging accuracy was calculated for the three approaches. PET/CT showed a sensitivity of 94% (95% CI, 86.1–98.3), specificity of 82% (95% CI, 72.2–93.3), positive predictive value (PPV) of 80%, and negative predictive value (NPV) of 95%. CT alone showed a sensitivity of 76% (95% CI, 63.0–81.0), specificity of 72% (95% CI, 63.0–81.0), PPV of 68%, and NPV of 86%. PET alone showed a sensitivity of 60% (95% CI, 51.0–69.0), specificity of 50% (95% CI, 40.0–60.0), PPV of 53%, and NPV of 66% (Table 5).

### 3.6. Cost-Effectiveness Analysis

Disease staging using CT alone correctly classified 73% of the study patients. The associated mortality associated with a lack of accuracy in disease staging was 2.7%. For PET alone these values were 57.7% and 2.8%, respectively. With PET/CT, disease was classified correctly in 89.8% of cases, and associated mortality fell to 1.4%. Thus, PET/CT shows greater disease staging accuracy and leads to reduced mortality. Medium-term survival was similar for all three alternatives.

CT, PET, and PET/CT achieved 3.739, 3.710, and 3.771 QALYs with a total cost of €16,877, €17,425, and €17,438, respectively. PET/CT led to savings by reducing the number of mediastinoscopies required to determine whether surgery was indicated, thus reducing the number of unnecessary procedures.

Disease staging by CT alone is superior to that of PET alone, which is associated with greater use of resources from the healthcare system and leads to more unnecessary procedures and fewer QALYs. With PET/CT, each year of life gained with respect to the use of CT alone would cost €45,374, and each QALY would cost €17,412.

**Figure 4:**
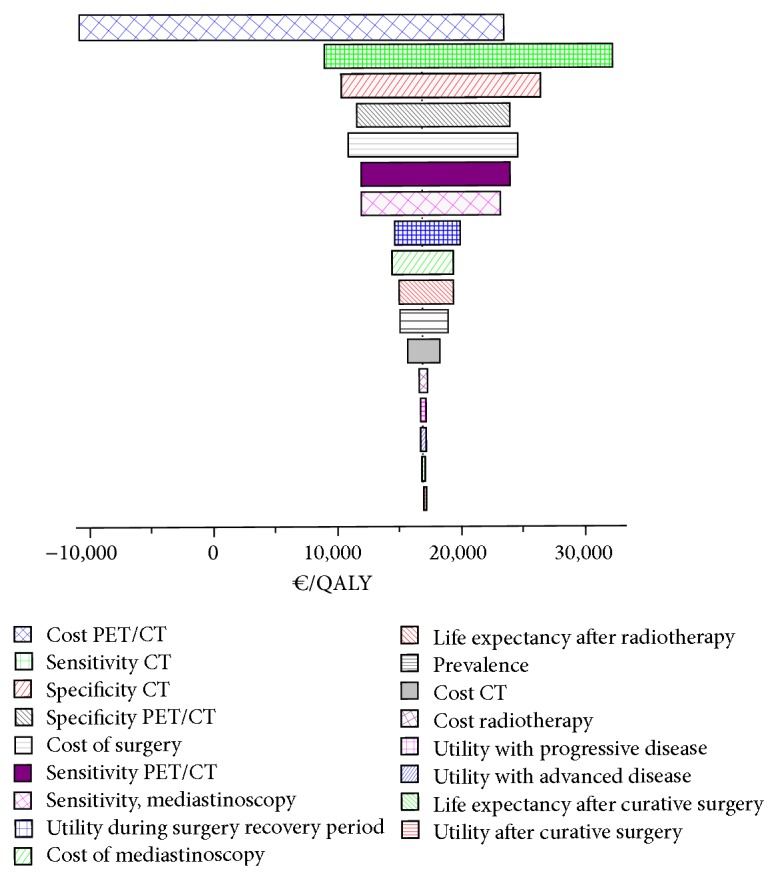
Tornado chart showing the variation in cost-effectiveness of PET/TC and CT depending on the variables included in the decision tree.

The tornado chart (Figure 4) shows the variation in cost-effectiveness of PET/TC and CT depending on the variables included in the decision tree.

The variable that showed the greatest influence in the incremental cost-effectiveness ratio was the low disease staging sensitivity of CT alone. This was associated with an incremental cost ratio ranging from €9,500 to €32,500 per QALY. The cost of PET/CT also had considerable influence; at a cost of €400, PET/CT would be superior to CT.

**Figure 5:**
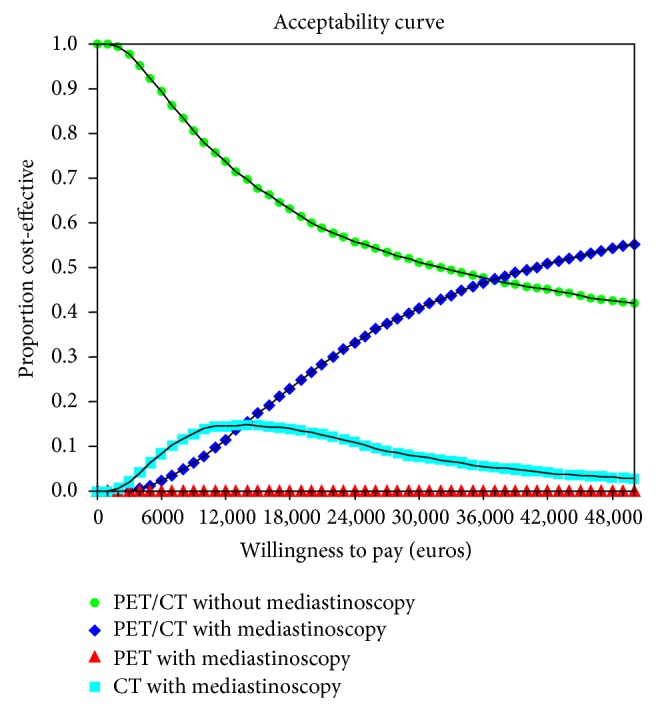
Cost-effectiveness acceptability curve.

The results of the cost-effectiveness acceptability curve can be seen in Figure 5.

## 4. Discussion 

The results of the present prospective study are consistent with those reported elsewhere [22–25]. PET/CT showed the highest sensitivity and specificity (94% and 82%, resp.) of the techniques assessed.

The PPV of PET/CT was 80% and the NPV was 95%. PET alone was the least accurate technique, owing to its poor anatomical resolution. All these studies reported significant differences between PET/CT and PET alone [22]. CT was more accurate in patients who underwent surgery in the present study than in the studies cited above.

The benefits of PET/CT over PET alone stem from the morphological information provided by CT, which improves detection of focal infiltration of the thoracic wall and the invasion of the mediastinum or vasculature. This information is not available with PET alone [22, 26]. CT performed using iodine contrast medium is, therefore, an important partner in this combined technique, enabling better characterization of the primary tumor and understanding of its relationship with the adjacent anatomical structures.

**Figure 6:**
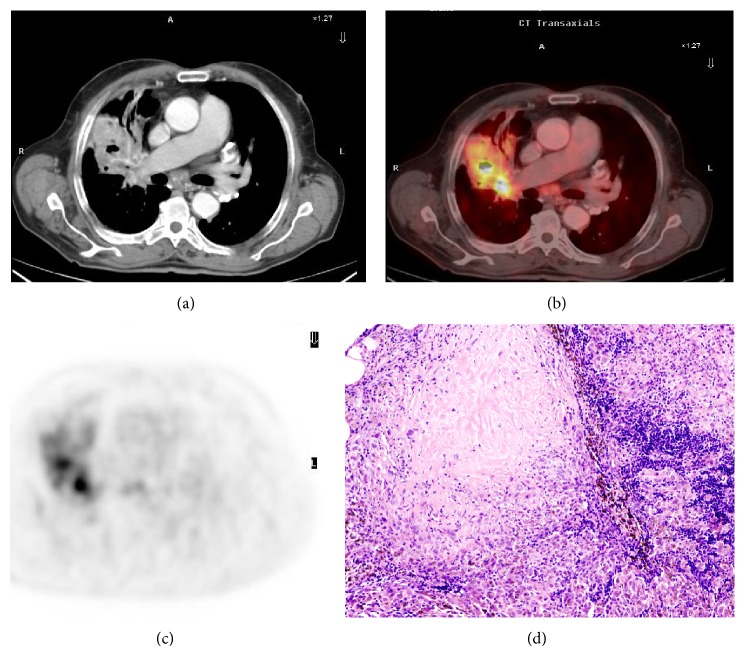
Images for a 62-year-old man with stage T2b and N1 NSCLC (epidermoid) in the right upper lobe. (a) CT image showing a lesion in the right upper lobe with adenopathy in space 7. (b) PET image showing ^18^F-FDG uptake (maximum SUV 9). (c) PET/CT image showing ^18^F-FDG uptake in the primary lesion and affected lymph node. (d) Histological analysis revealed the lymph node lesion not to be a tumor but rather hyperplastic anthracoid inflammation. Thus PET/CT provided a false-positive result.

PET/CT enabled better differentiation between hypermetabolic tumors and atelectatic lung parenchyma or areas of adjacent pneumonitis, as previously reported [27] (Figures 6(a) and 6(b)).

In lymph node disease staging, PET/CT provides good results with respect to histological examination (kappa = 0.756, *P* < 0.001). PET alone shows only moderate agreement (kappa = 0.566, *P* < 0.001), although detection of disease is less dependent on tumor size than CT alone, as reported by other authors [23, 24].

The overall sensitivity of PET has been reported to be 79–85% with a specificity of 89–92%. The values were significantly greater than those reported for CT alone (57–61% and 77–82% resp.) [28, 29]. However, both the sensitivity and specificity of PET and PET/CT vary with lymph node size; namely, they are very sensitive (100%) but less specific (78%) with large lymph nodes and fairly sensitive (82%) and specific (93%) with nodes of normal size [30].

The NPV of 90% reported for PET/CT in the literature [31] is a key finding. In contrast, PPV is much lower (70%), owing to false positives caused by inflammation. In our study, five false positives were recorded with PET/CT; four classifications of N1 and one of N2 were scored as N0 in the histology examination. These corresponded to nodes affected by anthracosis and/or lymphoid hyperplasia [30, 32]. False positives have been associated with a background of inflammatory disorders, such as tuberculosis, silicosis, and interstitial pneumonitis [32].

PET/CT results should be interpreted with caution [33]. The ACCP [34] recommends confirmation by histopathology in patients without distant metastases but with well defined, enlarged mediastinal nodes and in patients with normal-sized mediastinal lymph nodes and a central tumor. It is also recommended in patients with primary tumors in stage I but whose mediastinal lymph nodes show uptake in PET tests.

Yang et al. [32] showed that PET/CT correctly classified 81% of false negatives by CT (radiotracer uptake was detected in normal-sized lymph nodes) and 72% of false positives (lymph nodes of pathological size with no uptake). Twenty-seven of the patients included had metastatic disease: ten adrenal metastases, 15 generalised metastases (including liver and brain), and two bone metastases. Up to 40% of patients with NSCLC have metastases at diagnosis [35], mainly at the above-mentioned sites. Detection of these metastases is crucial when selecting treatment, since their presence rules out any curative intent.

**Figure 7:**
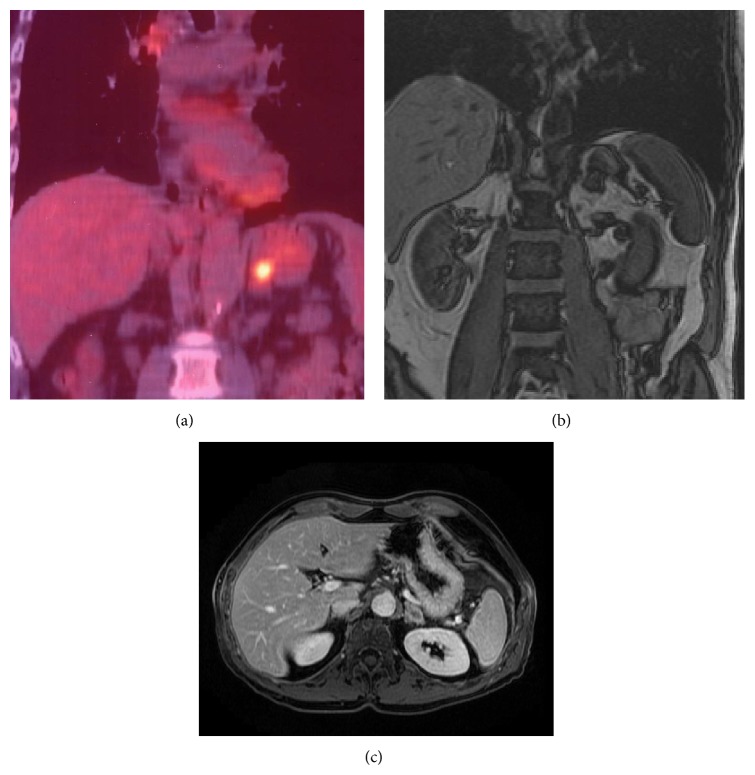
Images for a 70-year-old man with NSCLC (adenocarcinoma). (a) PET/CT showed intense ^18^F-FDG uptake by an adrenal metastasis. (b) Coronal out-of-phase image and (c) axial MR image with contrast medium confirming adrenal metastasis.

PET/CT enables more precise localization and better characterization of tumors scored as uncertain by CT alone [22, 23] and is useful in the assessment of adrenal nodules in patients with NSCLC, showing greater accuracy in the detection of metastases (84–92%) than PET, as well as greater specificity. The sensitivity of PET is close to 100% when the adrenal uptake of ^18^F-FDG is greater than that of the liver [35] (Figure 7).

The better disease staging achieved with PET/CT has been associated with changes in treatment in 9–15% of patients [23] in terms of curative intent, treatment type (chemotherapy, radiotherapy, or surgery), and planning of radiotherapy. In one randomized study [36], the inclusion of PET in the examination of patients with NSCLC led to a significant reduction in the number of unnecessary thoracotomies (from 41% to 21%).

The lack of a reference standard for PET/CT with respect to node involvement in nonsurgical patients is a limitation of this study. Histological examination of the anomalies detected by imaging methods in patients who are not candidates for surgery are currently performed using EUS and EBUS, which are included in the guidelines for staging lung cancer, mainly N2. As with the remaining techniques; they also generate false-positive results in lymph node stations that require mediastinoscopy [37]. A five-year follow-up revealed that none of the patients in stages IIIB and IV survived. The maximum survival was three years (one stage IIIB patient). However, the results obtained indicate that PET/CT should be the method of choice when staging disease in patients with NSCLC, given that complementary tests cannot provide more information.

The incremental cost-effectiveness of PET/CT was around €17,500 per QALY compared to CT alone. This figure is higher than that estimated by other healthcare systems [6–9], which considered PET and CT separately before analyzing their combined costs.

The present results showed that staging NSCLC by PET alone provided no benefit over staging by CT alone and required more resources. Our findings indicate that PET/CT is the most accurate strategy for disease staging. When willingness to pay is low (under €30,000 per QALY) [38], PET/CT is probably a more efficient strategy without mediastinoscopy, and treatment decisions can be adopted in accordance with the result. However, when willingness to pay is high (>€40,000 per QALY), PET/CT plus confirmatory mediastinoscopy is the most efficient strategy.

The strengths of the present study include its prospective design and the analysis of key variables (per life years gained and QALYs). This is the first cost-effectiveness analysis of these three techniques in Spain.

The cost-effectiveness analysis is limited in that it was based on costs obtained from the literature, which may not accurately reflect the costs associated with the Spanish Health System; unfortunately, local cost figures were not available.

In conclusion, PET/CT with intravenous contrast medium was found to be an accurate and cost-effective method for staging patients with NSCLC. Therefore, it should be the method of choice for staging in patients with this disease.

## Figures and Tables

**Table 1:** Variables used in the cost-effectiveness analysis.

Variable	Value	Lower limit	Upper limit	Source
Sensitivity of mediastinoscopy	0.72	0.63	0.81	[6, 7]
Specificity of PET	0.50	0.40	0.60	Present study
Specificity of CT	0.72	0.63	0.81	Present study
Specificity of PET/CT	0.82	0.75	0.89	Present study
Sensitivity of PET/CT	0.94	0.89	0.99	Present study
Sensitivity of PET	0.60	0.51	0.69	Present study
Sensitivity of CT	0.72	0.63	0.81	Present study
Prevalence	0.61	0.52	0.71	Present study
Costs				
Surgery cost	9917.83	7934.26	11901.37	DRG SNS [8]
PET cost	1091	872.8	1309.2	DRG CM [9]
PET/CT cost	1290	400	1500	DRG CM [9]
Radio + chemo cost	12807.33	10245.86	15368.80	DRG SNS [8]
CT cost	199	159.2	238.8	DRG CM [9]
Mediastinoscopy cost	4043.06	3234.44	4851.67	DRG SNS [8]
Outcomes				
Life expectancy after surgery	7.85	4.5	11.20	[10]
Life expectancy after radiotherapy	3.50	1.80	5.20	[10]
U after curative surgery	0.88	0.82	0.94	[6, 11]
U during surgery recovery period	−0.15	−0.30	0	[6, 11]
U with progressive disease	0.473	0.273	0.673	[12, 13]
U after palliative radiotherapy	0.673	0.65	0.70	[12, 13]

PET = positron emission tomography; CT = computed tomography; DRG = disease-related group; SNS = Spanish National Health system; CM = ‘‘Comunidad de Madrid” (Autonomous Region of Madrid); U = patient-valued utility with respect to disease stage.

**Table 2:** Concordance between the three diagnostic techniques and TNM staging (*n* = 63; 40 surgical patients and 23 with metastases).

	CT	PET	PET/CT	HP^*^ *gold standard *
IA	9	5	8	9
IB	17	11	8	11
IIA	0	2	1	0
IIB	7	3	8	6
IIIA	7	16	13	12
IIIB	2	5	2	2
IV	21	21	23	23
Kappa	0.694	0.614	0.836	
Stratified kappa	0.065	0.069	0.053	

HP: histopathology.

**Table 3:** Concordance between the three diagnostic techniques and histology.

		CT	PET	PET/CT	HP *gold standard *	Total biopsies
Size	T0	1	5	6	12	103
	T1	21	15	16	12	
	T2	39	37	41	39	
	T3	29	32	26	26	
	T4	13	14	14	14	

Kappa		0.726	0.596	0.882		

Stratified kappa		0.051	0.061	0.037		

Nodes	N0	59	39	43	24	40
	N1	8	19	16	4	
	N2	23	28	24	10	
	N3	13	17	20	2	
	N*x*				63	

Kappa (*n* = 40)		0.332	0.566	0.756		40

Stratified kappa		0.121	0.105	0.092		

Metastases	M0	81	78	78	80	25
	M1	22	25	25	23	

Kappa		0.8915	0.783	0.910		

Stratified kappa		0.048	0073	0.053		

HP: histopathology.

**Table 4:** Concordance of each diagnostic technique with the “T” of the surgical patients.

Site	CT	PET	PET/CT	HP *gold standard *
Left upper lobe	*k* = 0.90^*^	*k* = 0.70^*^	*k* = 1^*^	17
Right upper lobe	*k* = 0.64^*^	*k* = 0.56^*^	*k* = 0.73^*^	11
Middle lobe	*k* = 0.65^*^	No agreement	*k* = 1^*^	1
Lingula	*k* = 0.6^*^	No agreement	*k* = 1^*^	1
Left lower lobe	*k* = 0.95^*^	No agreement	*k* = 0.96^*^	5
Right lower lobe	*k* = 0.95^*^	No agreement	*k* = 0.96^*^	5

^*^ *P* < 0.001.

**Table 5:** Patient-based analysis of diagnostic accuracy of PET/CT, CT, and PET (*n* = 103).

	Sensitivity	Specificity	PPV	NPV
PET/CT	94 (86.1–98.3)	82 (72.2–93.3)	80 (ND)	95 (ND)
CT	76 (63.0–81.0)	72 (63.0–81.0)	68 (ND)	86 (ND)
PET	60 (51.0–69.0)	50 (40.0–60.0)	53 (ND)	66 (ND)

PPV: positive predictive value and NPV: negative predictive value.

ND: no data.
